# Infant Feeding and Infant Foods

**Published:** 1882-06

**Authors:** 


### Selections.

INFANT FEEDING AND INFANT FOODS. (Continued from page 526, May number.)

We are assured that Nestle examines his materials very carefully, uses only bread made of the very best wheat, and the crust only; and that the starch is transformed into dextrine and sugar, under a pressure of a hundred atmospheres, by overheated steam. Thus Nestle's food is not only the very best nutriment for infants, but also for mothers who would fain raise a supply of milk in their own breasts, though it appears that that would be an uncalled-for desire in the face of all that is said about Nestle's food being a perfect substitute. All of which is respectfully submitted by Mr. Nestle's prompter, Prof. Lebert, or, perhaps, rather by Prof. Lebert's prompter, and most of which is untrue.

Many years ago, fifteen or more, when Nestle and Lebert discharged their pamphlets, advertisements, and agents upon America, I was induced to try and employ Nestle's food. I could not say that in children of a few months or a year it did any harm; on the contrary, it was fairly tolerated by most of them. I consented even to add my name to those of others recommending the article—the only time I ever did so in my life. A few years later, another advertisement appeared, which advised rather against the purchase of the former preparations, and recommended the recent modification. It was then, at last, that I resolved to scrutinize what I had recommended in good faith without any examination. I found, 1, that the contents of a number of boxes did not closely resemble each other; 2, that the main substance was made up of starch; 3, that even the cell membranes were not equally crushed, and a great many not changed at all. It has also been found, and urged mainly by Eliza McDonogh (*Inaug. Diss.*, Zurich, 1877,) that the fragments of the microscopic elements exhibit the appearance of mechanical destruction, and not that brought about by heat. J. Miescher also states that he finds no essential difference between Nestle and common flour. Iodine yields instantly its blue reaction; the starch elements are partly free, partly still locked up in their cells. Some particles are albuminous; they turn yellow with iodine. In his experiments the watery extract was alkaline; in those of McDonogh it was acid, and contained but little dextrine. Cnyrim found in the boxes examined by him, sugar and dextrine to the amount of 45.56 per cent., but still 20.76 per cent. of starch was left without any change whatsoever. And the result of Jacobsen's examinations is that the contents of the boxes are not alike.

Gerber, also a large advertiser, and Nestle's Swiss rival in America, claims to have improved Nestle's flour by a difference in the process of preparation; the methods of which, however, are not communicated. His flour is wheat, which is again praised as the one article containing all the material required by the growing body in the smallest possible bulk. The same enumeration of the elements is made of an organic salt, and their mixture with organic substances is spoken of as if it were a new discovery. Potassium and phosphatic combinations, albuminates, carbon hydrates, fats, are whirled about in the most impressive and bewildering manner by every one who has a cheap article for sale at a high price. The greatest stress is always laid on the presence in the article offered for sale of all the chemical elements necessary for the building up of the body. But that presence alone, I repeat, is not sufficient; chemical figures and equivalents still enjoy too much respect and a sort of superstitious veneration. A thorough preparation preliminary to digestion is required, though the proportion of elements in a mixture were ever so appropriate. In regard to wheat, however, this proportion is not the very best at all, for wheat contains more starch than do barley or oatmeal.

In regard to both of them, Nestle and Gerber, Drs. Altherr and Lorch, in a little book entitled " On the Weighing of Infants for the purpose of Determining the Nutritive Value of Woman's Milk, Cow's Milk, Nestle's and Gerber's Flours, and Liebig's Soup," Erlangen, 1877, has no great praise to bestow. Both are declared to be improper foods for the newly born and infants in the first few months; but they admit in the later months of life, when farinaceous foods of all kinds are well tolerated, they also may be given in the same manner. Similar observations have been made in the Child's Hospital, at Berne, Switzerland; and similar remarks are due to the Anglo-Saxon Food, which contains milk, some gluten, and plenty of starch, like the others.

In all of the twenty-two European infant foods examined by Eliza McDonogh, the starch was never changed to such a degree as to render the recognition of its source impossible. Wheat, corn, rye, barley, could always be recognized under the microscope. A number of the granules were broken up, it is true, but many more were intact. Thus, not even the very exact and uniform distribution of the starch is accomplished. All of them consist of baked flour of some kind, either by itself or mixed with sugar, milk, or salts. There are some, however—for instance, that of Hartenstein—consisting of wheat and oats, which has not even undergone the baking process.

Part of these European preparations have been naturalized with us. Some have made a great reputation, and probably money for many beneficent manufacturers, agents, and salesmen. But they are not alone in the field. Saul slew a thousand, David ten thousand. Europe has twenty odd infants' foods; America more than twice that number. Infants must no longer complain of lack of interest roused in their behalf.

Nature is the same all over; the nature of all natural things alike; that of wheat, oats, and manufacturer, wherever they be found. Thus the same results in regard to the composition of "infant foods" are reached here as in Europe. But a few days ago I was pleasantly surprised by the brief statements of Dr. E. Cutter, who, for *Gaillard's Medical Journal* (Jan., 1882), has undertaken the task of studying (microscopically) all the infant foods in our market. His researches, naturally, refer mainly to the proportion of gluten to starch. And some of his statements I shall refer to here, desiring to give them the greatest possible publicity. I wish this brief article of his could be distributed in a hundred thousand copies, reprinted in every secular paper, read from every platform and pulpit of the land. For it is time that fraud should be stopped, and a nefarious trade suppressed.

Amongst the better preparations are Mellin's Food. It claims to be the only substitute for mother's milk, and not farinaceous. It is not the only substitute, but the starch is converted into dextrine, as in Horlick's Food.

Franklin Mills Wheat Flour contains more gluten than most of the rest. So does Arlington Wheat Meal, which is ground coarsely, and contains much gluten. Also Hawley's Liebig's Food, which contains wheat gluten cells, barley gluten cells, barley tegument, wheat starch, and cooked granular masses, which do not polarize light.

Mead's Malted Wheat Flour, a coarse flour or meal, which does not polarize light well ; a good preparation.

Baby Sop is made of unhulled oats, malted and crushed, and contains all its elements ; gluten cells of characteristic shape, and starch grains smaller than those of wheat.

As you descend the ladder, there is Keasbey & Mattison's Infants' Food. No gluten cells ; consists of grape sugar and dextrine, and claims to contain alkaline phosphates.

Mother's Breast-milk Substitute is better than its ignorant advertisements ; it contains gluten cells, bundles of wheat starch, barley starch, and gluten granules.

Imperial Granum, the salvator for invalids and the aged, an incomparable aliment for the growth and protection of infants and children, a superior nutriment in continued fevers, and a reliable remedial agent in all diseases of the stomach and intestines ; that which makes strong bone and muscle ; that which makes good flesh and blood ; that which is easy of digestion, never constipating ; that which is kind and friendly to the brain ; and that which acts as a preventive of those intestinal disorders incidental to childhood—Imperial Granum food, which claims that the starch, impurities, and soluble matter are effectually excluded, and that the gluten only is retained, *shows no gluten cells under the microscope*, and is no better than it should be, no better than common flour, which, when fine and white, is deprived of three-fourths of its gluten, and no better than Savory & Moore's Food, which is also like common flour.—(E. Cutter.)

*Ridge's Food* is advertised as "a perfect food for infants." Ephraim Cutter tells you how perfect a food it is. It contains the beard of wheat, wheat starch mass, starch bundles, apparently of maize, caked mass of starch which does not polarize light, starch grains, starch granules, and small gluten granules. And he adds: "The proprietors must add gluten cells, at least in the proportion found in wheat or maize, to bring the product up to the standard of wheat flour."

*Crosby's Brain and Nerve Food*, which claims to be composed of vitalized phosphates from ox-brain and wheat-germ, consists almost exclusively of starch. There are no characteristic gluten cells, no nerve-fiber, no axis cylinder fiber, no ganglion, nor multipolar cell. The advertisement claims that the brain is that of the ox, but the label states that the brain is that of the fish. Label advertises 730 parts of starch in 1000, claims some gluten; if this exists, it is the granular gluten of common flour. There are 270 parts of so-called vitalized salts asserted to be in this food; that is, salts in connection with the organic substances named. If this be so, all the albuminoids in all the foods in the market are vitalized, also. There is but little gluten, if any, in this food. (E. Cutter.)

*Blanchard's Glutena*, 90 per cent. of starch, 10 of gluten, the reverse, exactly, of the claim made for the food.

*Blair's Wheat-food*, abundance of starch, no gluten.

*Redmond's Cerealine*, starch, very little gluten.

*Durkee's Glutena*, wheat, almost exclusively starch.

*Farwell's Gluten Flour* is advertised as "gluten left behind after the starch is blown out," contains starch, almost no gluten.

*Victor's Baby Food* is *said* to "resemble mother's milk closely," and *is* like cracker and biscuit ground up.

*Taylor Brothers' Pure Bermuda Arrow-Root* consists of potato starch, with an occasional addition of arrow-root.

*Hubbell's Prepared Wheat*, almost exclusively of starch.

No gluten is contained in *New York Food Company's* cold blast flour, barley flour, buckwheat flour, India wheat flour, Lost Nation wheat flour, common Minnesota flour, Hazleton flour,

Puritan flour, Patapsco flour, Underwood flour, fine granulated wheat flour.

*Gluten Flour, New York Health Food Company*, is common flour. The most superficial examination exhibits starch in abundance.

After all this, with all my heart I assent to E. S. Gaillard's editorial remarks, which are as follows:

"It is almost criminal that such great questions, affecting the health, and, therefore, the happiness and wealth of a nation, should be left to the ignorance of the miller and the baker; to the foolish customs of society, to the equally foolish test of the mere appearance of bread, and to the fashionable restaurants and hotels. Manufacturers of foods for the sick, and, above all, for infants, should be held to the strictest accountability. It is the highest duty of the physician to learn the facts in regard to meal foods, and to use his information for the benefit not only of the sick, but of the whole community." And experience shows, that as the committee report referred to expressed it last year, the community insists, with the utmost pertinacity, upon giving their babies, as soon as weaning time arrives, or before, such articles of food as they know nothing about. When an adult sits down to a meal and finds placed before him articles of food with which he is not familiar, he makes inquiries in regard to such articles before eating them. The baby, however, is credulously fed upon things with which the child, father, mother, or doctor has not the least familiarity; all of these foods which are sold in large quantities have a composition which is unknown to the public. When a manufacturer designs to say anything about his merchandise, it is to the effect that the food offered is the best in the market, that it is the proper thing and only thing for children and invalids of all ages, that the relation of the albuminous substances to carbo-hydrates is exactly correct, and that a package costs a certain amount of money. In regard to this subject the public appear to be smitten with absolute blindness. They insist upon forgetting that the man who offers for sale, and advertises at a very heavy expense, does so, as society is constituted, for his pecuniary advantage solely. To say that when the article offered is not good it will find no market, is deceiving yourselves, experimenting on your baby, relying on the character of a single man or corporation, on the honesty or intelligence of the manufacturer's chemist, or his superintendent, or his workmen, on the nature and condition of the elements used in the composition of the article, and on ever so many influences, which can work before the manufactured article gets into the hands of the consumer. Why the sellers and advertisers of unknown compounds should be more trusted than those who raise and sell a simple article of food, such as milk, which is constantly adulterated, can hardly be perceived. Is it necessary to say that the factory furnace is lighted more in the interest of the proprietor than for the benefit of the public ?

Still, in regard to the growing evil, which has assumed vast proportions, the profession is at fault, to a certain extent. There are but few who are aware of the inexpediency and sometimes danger attending the exclusive feeding of cow's milk, and look for substitutes. Examples of infants' thriving on almost any food are numerous; the public taste runs in the direction of the unknown; thus the responsibility of advice or assent is but a slight one; many of the foods in the market come in a pleasant form and convenient for use; thus the food-business firm certainly thrives. Professional men have become used to look upon the sale of patented foods as something quite unobjectionable. Those imbued with the strictest sense of ethics, who would not patent an invention, nor tolerate the fellowship of a professional man who so does, who frown upon patented medicines, because they are unknown and unknowable compounds, who object to reducing medical science to a mercenary standpoint, these very men forget their habits and principles when the question of patent-right and secrecy comes up in regard to patent foods. If I add, that many of the scientific journals of Europe, particularly those in Germany, dedicated to the study of children's diseases, are frequently used for the purpose of discussing the merits and effects of some new infant foods, it is only to show to what extent the evil had grown.

No profound thinking is required to appreciate the fact that a great many of the articles offered for sale are unmitigated frauds; and that a few are available compositions. But the very fact that they are compositions, that everything organic may spoil, that every compound depends on too many circumstances which are apt to interfere with its uniform condition, and that when you rely on a compound, you rely at the same time on a proprietor, his foreman, his workman, his chemist, you feel that you are easily deceived or disappointed. Besides, for an article, the constituents of which you can purchase at a low price, you are taxed to an inordinate extent.

And why all this? Because cow's milk is found to be defective, when compared with mother's milk. These defects I have pointed out. It contains too much fat, and too much caseine, the latter of abnormal condition. Instead, however, of correcting the faults of milk, the latter is simply superseded and exchanged for something else. Is potato starch or even wheat, more similar to mother's milk, than is that of the cow? Now the proportions of fat and caseine can be regulated by the addition of water; but dilution is not the only function of the water added to the milk; the digestibility of the latter is also increased.

When albuminous material is subjected to artificial digestion with pepsin, and the process ceases, it recommences when more water is added. When the amount of water in the mixture is large, digestion will take place in a lower temperature than without it. Many digestive disorders in the living are due to lack of water, particularly in infants, who seldom or never are supplied with it in addition to what they obtain in their regular food. Infants who are apt to perspire much, either well or rachitic, who are kept in warm beds most of the time, who are exposed to summer heat, and lose much moisture through their skin, ought to be offered water a great many times in the course of the day. Particularly fat infants, who are mostly developing a rachitic or scrofulous disposition, ought to drink plenty of water. It accelerates tissue changes, and, therefore, animals which are to be fattened are habitually deprived of water. The rule then holds good in infant alimentation, that food, whatever it be, shall be given in considerable dilution.

But in the face of all the advantages water has, it is by itself not the proper dilution for the caseine of cow's milk. Some admixture must be found which is apt to overcome the dense coagulations of caseine referred to before. The particles of caseine must not be permitted to remain in juxtaposition, but separated from each other to such an extent that coagulation in the stomach will take place in small loose flakes of the kind presented by curdling mother's milk. This object is accomplished by all farinaceous substances, without regard to their large or small percentage of starch; also by gum arabic and gelatine. Of the farinaceous substances, I have but two to recommend, barley and oatmeal. And for good reasons. The starch they contain is more easily dissolved and transformed by saliva than that found in wheat, which is the stock and trade of the infant-food manufacturers. And, moreover, there is less of it in them than in wheat.

The barley and oat-meal are the two substances I mostly employ, as their chemical constituents are nearly alike, with the exception of a large portion of fat in oat-meal, which is not found in barley. Barley water, or thinned and sweetened oatmeal gruel, may be given to the child, even at the breast. The indications for the use of one or the other lie in the condition of the infant. Where there is a decided tendency to constipation, I prefer oat-meal; where there is no such tendency, as usual, or perhaps even a tendency of the bowels to be loose, I employ barley. The "prepared" barley is a good preparation for older children only, for the whiter it is the larger is its percentage of starch. It is safest, as no mistake or deception can then take place, for every mother to grind the whole barley in a common coffee-grinder of her own. The younger the baby the more advisable does it become to boil whole barley for hours, to secure the contents of the bran. A teaspoonful of either is boiled in from five to eight ounces of water, with some salt, for twelve or fifteen minutes, the decoction to be quite thin for young infants, thicker for later months, and then strained through a linen cloth. Infants of four or six months are to have equal parts of this decoction (which ought to be made fresh for every meal), and of boiled cow's milk; some sugar besides. At an early age, the thin decoction, at a later, the milk ought to prevail in the mixture, which ought to be given at a temperature of 80–90°. The newly-born, which has to wait some days for his spring to open for him, is best fed with a mixture of from four to (in summer) six parts of thin barley water, and one part of boiled milk. A baby of two months will take the same mixture in the proportion of from two or three to one.

I beg leave to quote a single author. Biedert, a conscientious worker, the author of competent contributions to medical science, and particularly to infant hygiene, himself the inventor of a cream mixture for the use of well and sick infants, expresses himself in the following manner in the very circular accompanying his preparations: "The first demand on a method of nourishing infants must be this—that the nutriment is tolerated by all infants, including the feeble and sick, in a manner similar to mother's milk. Now I have found that a food coming up to this requirement, must not contain more than 1 per cent. of caseine. Accordingly, cow's milk must be diluted with an indifferent thin decoction (oat-meal or barley.) In the beginning, the dilution must be in the proportion of 1 to 3 or 4; this admixture must be gradually reduced. This is the very mixture which surpasses all compounds thus far recommended, and all of the artificial preparations." And still in the face of this testimonial, than which I do not wish a more positive one, he invents a cream mixture.

Though my remarks must necessarily draw to an end, I must not close without at least one additional word in regard to some other constituent of infant food. Beside sugar, which is required both by taste and physiological necessities, the addition of common table-salt in moderate quantities both to cow's milk and to farinaceous substances is advisable.

The addition of chloride of sodium to the food is the more important the more the milk is mixed with a vegetable decoction. Carnivorous animals crave no salt; herbivorous, however, a great deal, although in their food the absolute amount of chlorine and sodium is as large as that of the former. They are easily drawn by hunters into fields and woods strewn with salt; and the instinct of mankind has early taught how to add more salt to herbaceous than to animal food.

We find that the craving for salt grows in proportion to the amount of potassium salts contained in the food. In that of herbivores there is twice or even four times as much potassa compared with its chloride of sodium as in that of carnivores; potatoes. with their large amount of potassium, combined with chlorine and phosphoric and citric (pomic) acids, necessitate the addition of large quantities of chloride of sodium. In the food of carnivores, however, who eat whole animals, one equivalent of potassium is nearly balanced by one of sodium and one of chlorine.

Potassium salts and soda salts both exist in the blood, the former principally in the blood corpuscles, the latter entirely in the serum or watery part of the blood. There is chloride of potassium in both corpuscles and serum, but phosphate of potassium in the corpuscles alone. Now if there should ever be in the blood more phosphate of potassium than can be taken up by the corpuscles, it will be immediately decomposed by the chloride of sodium in the serum—chloride of potassium and phosphate of sodium are formed, and these are both rapidly eliminated by the kidneys, and pass away in the urine. By this means, chloride of sodium is carried away, and must be replaced; in other words, vegetable food requires the addition of a great deal of salt.

The substitution of cow's milk for woman's milk necessitates the addition of salt for a similar reason; for while 1000 parts of human milk contain only 0.70 chloride of potassium, 1000 parts of cow's milk contain 1.30.

There may be infants—I have been told of more cases than I have seen myself—who do not bear barley. Others may not bear oat-meal. If so, or if for some reason or other it is wise or whim to discontinue them, it must not be forgotten that, though feeding a breastless child without cow's milk, appears preposterous, feeding with cow's milk alone is dangerous. I do not permit it. I want an admixture at all events capable of diluting, as it were, the caseine. For that purpose, and for that alone, I formerly recommended gum arabic or gelatine. However, our knowledge of these substances has since improved. Prof. Uffelmann, in Rostock, had a little patient, otherwise healthy, who carried a gastric fistula. Through a large opening in his abdominal and stomach wall, he experimented on the child's digestion. Unmistakable and numerous observations taught him that both arabic gum and gelatine are not only emollient and soothing, but diretly nutritious. Thus the list of those substances which may be added to boiled cow's milk, to make it available as the regular food for infants, is by no means small. They all come up to the requirements we look for in such substances. They must be perfectly simple and recognizable. They must be accessible, and for sale everywhere. The mode of preparing them must be perfectly simple and easy. They must be cheap.

They must be as nearly the substitute of mother's milk as an imitation can be like the original. They must be alike for throne and den, for Fifth Avenue and Mulberry Street, for the wet-nurses for the throne and for Fifth Avenue, come also from the dens and from Mulberry Street. It is not wise to forget that nature is republican in principal and democratic in practice. If too often there be no equality before a court of so-called justice, there is equality before that of physiological law.—*Med. News.* Feb. 18, 1882.

